# Efficiency and effectiveness of the use of an acenocoumarol pharmacogenetic dosing algorithm versus usual care in patients with venous thromboembolic disease initiating oral anticoagulation: study protocol for a randomized controlled trial

**DOI:** 10.1186/1745-6215-13-239

**Published:** 2012-12-13

**Authors:** Antonio J Carcas, Alberto M Borobia, Marta Velasco, Francisco Abad-Santos, Manuel Quintana Díaz, Carmen Fernández-Capitán, Nuria Ruiz-Giménez, Olga Madridano, Pilar Llamas Sillero

**Affiliations:** 1 Clinical Pharmacology Department, La Paz University Hospital, Pharmacology Department, School of Medicine, Universidad Autónoma de Madrid, IdiPAZ, Paseo de la Castellana, 261, 28046, Madrid, Spain; 2 Pharmacology Department, School of Medicine, Universidad Autónoma de Madrid, Madrid, Spain; 3 Clinical Pharmacology Service, La Princesa University Hospital, Pharmacology Department, School of Medicine, Universidad Autónoma de Madrid, Instituto de Investigación Sanitaria Princesa (IP), Madrid, Spain; 4 Emergency Department, La Paz University Hospital, School of Medicine, Universidad Autónoma de Madrid, IdiPAZ, Madrid, Spain; 5 Internal Medicine Department, La Paz University Hospital, School of Medicine, Universidad Autónoma de Madrid, Madrid, Spain; 6 Internal Medicine Department. La Princesa University Hospital, Instituto de Investigación Sanitaria Princesa (IP), Madrid, Spain; 7 Internal Medicine Department, Infanta Sofía Hospital, Madrid, Spain; 8 Department of Hematology, Fundación Jiménez Díaz Hospital, Madrid, Spain

**Keywords:** Pharmacogenetic, Acenocoumarol, Hematology

## Abstract

**Background:**

Hemorrhagic events are frequent in patients on treatment with antivitamin-K oral anticoagulants due to their narrow therapeutic margin. Studies performed with acenocoumarol have shown the relationship between demographic, clinical and genotypic variants and the response to these drugs. Once the influence of these genetic and clinical factors on the dose of acenocoumarol needed to maintain a stable international normalized ratio (INR) has been demonstrated, new strategies need to be developed to predict the appropriate doses of this drug. Several pharmacogenetic algorithms have been developed for warfarin, but only three have been developed for acenocoumarol. After the development of a pharmacogenetic algorithm, the obvious next step is to demonstrate its effectiveness and utility by means of a randomized controlled trial. The aim of this study is to evaluate the effectiveness and efficiency of an acenocoumarol dosing algorithm developed by our group which includes demographic, clinical and pharmacogenetic variables (*VKORC1, CYP2C9, CYP4F2* and ApoE) in patients with venous thromboembolism (VTE).

**Methods and design:**

This is a multicenter, single blind, randomized controlled clinical trial. The protocol has been approved by La Paz University Hospital Research Ethics Committee and by the Spanish Drug Agency. Two hundred and forty patients with VTE in which oral anticoagulant therapy is indicated will be included. Randomization (case/control 1:1) will be stratified by center. Acenocoumarol dose in the control group will be scheduled and adjusted following common clinical practice; in the experimental arm dosing will be following an individualized algorithm developed and validated by our group. Patients will be followed for three months. The main endpoints are: 1) Percentage of patients with INR within the therapeutic range on day seven after initiation of oral anticoagulant therapy; 2) Time from the start of oral anticoagulant treatment to achievement of a stable INR within the therapeutic range; 3) Number of INR determinations within the therapeutic range in the first six weeks of treatment.

**Discussion:**

To date, there are no clinical trials comparing pharmacogenetic acenocoumarol dosing algorithm versus routine clinical practice in VTE. Implementation of this pharmacogenetic algorithm in the clinical practice routine could reduce side effects and improve patient safety.

**Trial registration:**

Eudra CT. Identifier: 2009-016643-18.

## Background

Antivitamin-K oral anticoagulants, which include acenocoumarol, are highly effective drugs for the treatment of venous thromboembolism (deep venous thrombosis or DVT, and pulmonary thromboembolism or PTE), atrial fibrillation and patients with mechanical heart valves [1]. However, hemorrhagic events are frequent [2,3] due to the narrow therapeutic margin of these drugs and the considerable interindividual variability in the pharmacokinetics and pharmacodynamics of these drugs.

Studies performed with warfarin and acenocoumarol have shown that the relationship between the genotypic variants *CYP2C9* and *VKORC1* account for approximately 30% to 40% of the variability in the response to these drugs [4-6] while various clinical factors explain between 15% and 20% of the variability. More recently, other polymorphisms have been identified that influence the doses of warfarin and acenocoumarol necessary to maintain a stable international normalized ratio (INR). The *CYP4F2* will hydroxylate the side chain as the first step in the inactivation of vitamin E, although its role in the vitamin K/acenocoumarol pathway is not yet known [7-10]. Apolipoprotein E (ApoE), in turn, mediates the uptake of lipoproteins rich in vitamin K by the liver and other tissues [11,12].

Once the influence of these genetic and clinical factors on the dose of acenocoumarol needed to maintain a stable INR has been demonstrated, new strategies need to be developed to predict the appropriate doses of this drug. Several pharmacogenetic algorithms have been developed for warfarin [13-22] and three have been developed for acenocoumarol. The first algorithm for acenocoumarol [23] is based on a score generated with genetic variants of *CYP2C9* and *VKORC1*. The second algorithm was published by the EU-PACT group [24] and includes clinical (age, gender, weight, height and use of amiodarone) and genetic variables (*CYP2C9* and *VKORC1*). Our research group has developed a third algorithm for individualization of the acenocoumarol dose in patients with VTE using a multiple linear regression analysis in a derivation cohort (n = 117) that includes clinico-demographic variables (age, body mass index, use of amiodarone and enzyme inducers) and polymorphisms of the *VKORC1*, *CYP2C9*, *CYP4F2* and ApoE genes [25]. The clinical factors explained 22% of the dose variability, which increased to 60.6% when pharmacogenetic information was included (*P* <0.001). In the testing cohort (n = 30), clinical factors explained a 7% of the dose variability, compared to 39% explained by the pharmacogenetic algorithm. The pharmacogenetic algorithm correctly predicted the stable dose in 59.8% of the cases (derivation cohort) versus only 37.6% predicted by the clinical algorithm (95% CI: 10 to 35). We have implemented this algorithm in a public web page (http://www.acenocoumaroldosing.com).

After the development of a pharmacogenetic algorithm, the obvious next step is to demonstrate its effectiveness and utility by means of a randomized controlled trial. One such trial in the USA is being performed with warfarin: NCT01178034 (ClinicalTrials.gov), and includes patients with atrial fibrillation. Another trial has been completed: NCT00511173, which included patients with atrial fibrillation, pulmonary embolism and deep vein thrombosis. In Europe, there is an ongoing clinical trial to test whether the dosing algorithms for coumarin anticoagulants (including acenocoumarol) improves the clinical outcomes for patients [26].

Our group proposes this randomized clinical trial that compares the standard dosing of acenocoumarol with the adjustment based on the developed acenocoumarol dosage algorithm (Eudra CT: 2009-016643-18).

## Methods

### Design and setting

**Figure 1:**
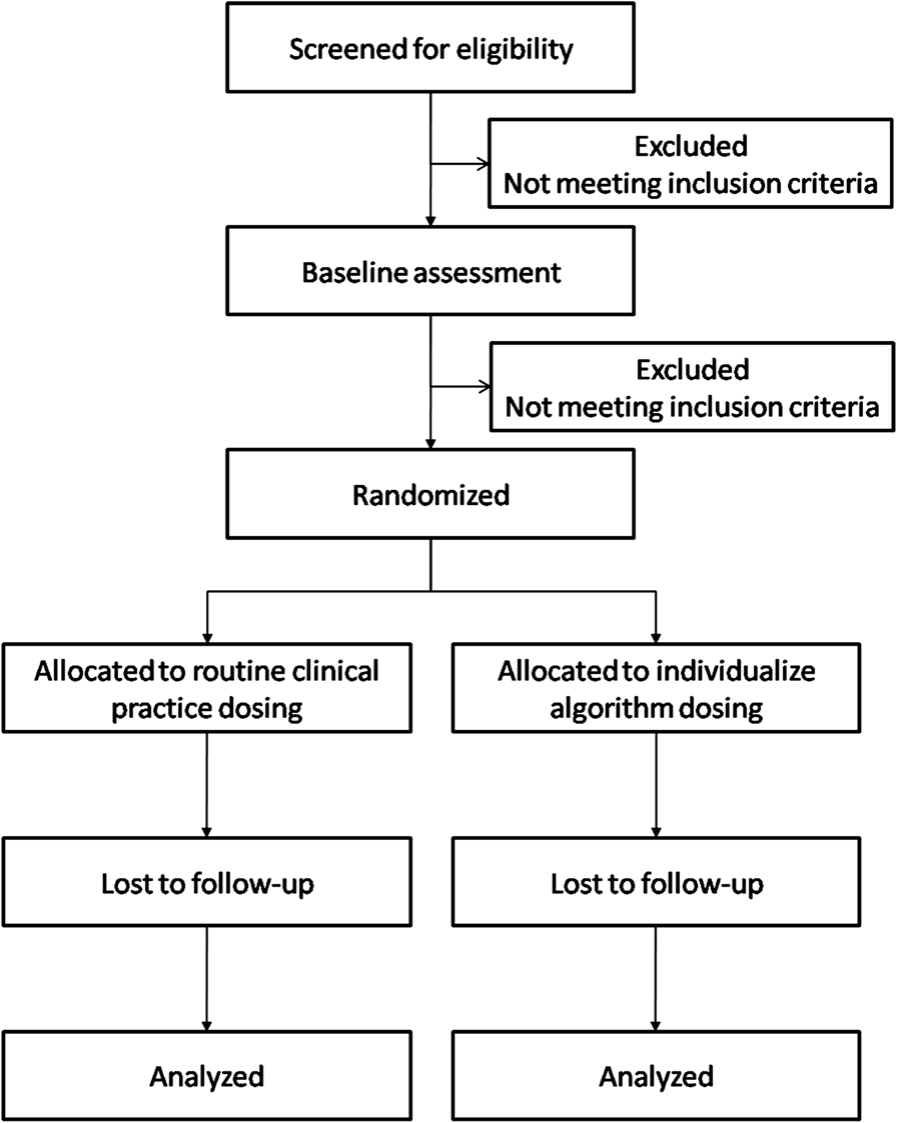
Flowchart of study procedures.

The clinical trial was designed as a pragmatic, randomized, parallel two-arm, single blind trial to compare the individualized adjustment of acenocoumarol dosage using a pharmacogenetic algorithm versus the standard adjustment, in patients initiating oral anticoagulation for the treatment of venous thromboembolism. The follow-up period will be three months (Figure 1). The study will be performed in five separate hospital centers in the Community of Madrid (Spain). The recruitment period started in March 2011 and is scheduled to end in the fourth quarter of 2012.

### Study population

The target population is patients with new diagnoses of pulmonary thromboembolism and/or deep vein thrombosis diagnosed using objective imaging tests (CT angiography, venous Doppler ultrasound), who require anticoagulant therapy with acenocoumarol and who meet the following inclusion criteria and none of the exclusion criteria.

#### Inclusion criteria

• Male and female patients who are diagnosed with VTE (PTE and/or DVT) who will start anticoagulant therapy with acenocoumarol.

• Target INR between 2 and 3.

• Over 18 years of age.

• Women of childbearing age should take effective contraceptive measures (barrier methods) to avoid pregnancy during the treatment with acenocoumarol.

• Subjects may give their written consent to participate in the study once they have received information on the design, goals and possible risks of the study and are made aware that they can withdraw from the study at any time.

#### Exclusion criteria

• Patients anticoagulated with acenocoumarol at the time of inclusion (≥2 doses).

• Pregnant or breastfeeding women.

• Patients with renal failure (creatinine clearance ≤30 ml/min).

• Patients with advanced liver failure (stage C of Child-Pugh).

• Patients with a life expectancy of less than six months.

### Definition of the intervention

Once included in the clinical trial, patients will be randomly assigned to the experimental or control groups. Initially, all patients will receive treatment with low molecular weight heparins (LMWH) along with the standard acenocoumarol dose established according to age, for a maximum of three days (the time required to obtain the genotyping result). Subsequently, dose adjustment will be different in the two groups:

Patients assigned to the experimental strategy will receive the regimen determined by the pharmacogenetic algorithm, which includes demographic (age, gender, weight and height), clinical (concomitant medication) and pharmacogenetic (polymorphisms of *CYP2C9*, *VKORC1*, *APOE* and *CYP4F2*) variables [25]. INR is also taken into account.

Patients assigned to the control strategy will receive doses according to the standard procedure in routine clinical practice.

As a guide for handling and adjusting the acenocoumarol dosages, the researchers will be referred to the local guidelines developed in ‘Area 5’ for the management of patients treated with acenocoumarol.

### Objectives

#### Primary

To evaluate the effectiveness of an acenocoumarol dosing algorithm that incorporates demographic, clinical and pharmacogenetic variables of patients with venous thromboembolism.

#### Secondary

• To evaluate the safety of the incorporation of genetic data in the dose adjustment, by comparing the frequency of appearance of relevant clinical events in patients whose dosage is adjusted by means of the designed algorithm versus standard clinical practice.

• To evaluate the efficiency of implementing an acenocoumarol dosing algorithm that incorporates pharmacogenetic variables. To accomplish this, we intend to perform an economic analysis of the incorporation of the genotypic determinations versus standard clinical practice.

### Assessment variables

#### Effectiveness variables

##### Primary

• Percentage of patients with an INR within the therapeutic range (INR between 2 and 3) on day seven after the initiation of treatment with acenocoumarol.

• Time from the start of the oral anticoagulant therapy to get a stable INR within the therapeutic range (INR within the therapeutic range for three consecutive measurements for at least two weeks, with a maximum difference between the daily average doses of 10%).

• Number of INRs within the range in the first six weeks of treatment.

• Number of INRs within the range during the three months of the study.

##### Secondary

• Proportion of time within the therapeutic range, using a linear interpolation method [27].

• Number of special INR measurements (those that are performed in addition to the scheduled measurements in the program of visits established in this protocol: days 0, 3, 7, 15, 30, 60 and 90).

##### Safety

• Proportion of patients with relevant adverse events during the first three months following the start of oral anticoagulation. These events include the following: INR >4 or <1.5; need for vitamin K; bleeding episode; thromboembolic event; need for LMWH; acute myocardial infarction; acute stroke; and death from any cause.

##### Pharmacoeconomic evaluation

• The pharmacoeconomic evaluation will be performed according to a parallel and independent protocol with special consideration of the direct costs associated with the health care of these patients.

### Study procedures

The various study visits will be distributed as shown in Table 1:

**Table 1:** Study timeline

**Day**	**−3 or −2**	**0**	**3**	**7 (±1)**	**15 (±3)**	**30 (±5)**	**60 (±7)**	**90 (±7)**
Recruitment	**√**							
Informed consent	**√**							
Selection criteria	**√**							
Medical history	**√**							
Physical examination	**√**							**√**
Blood samples for pharmacogenetics	**√**							
Randomization	**√**							
Acenocoumarol dosing (initiation/adjustment)^a^		**√**	**√**	**√**	**√**	**√**	**√**	**√**
INR determination		**√**	**√**	**√**	**√**	**√**	**√**	**√**
Evaluation of side effects		**√**	**√**	**√**	**√**	**√**	**√**	**√**

^a^In order to perform appropriate dose adjustments, additional visits can be scheduled as needed, at the discretion of the clinical researcher.

Preselection phase: The patients with VTE will be identified in the emergency department or hospital ward within the first 48 hours of the VTE diagnosis (PTE and/or DVT) and will be checked to ensure they meet the selection criteria.

Before any specific study procedure is performed, the patient will receive the information verbally and in writing through the patient information sheet. The patient must consent to participation in the study in writing (two copies). The patient will keep a copy of the information sheet and the informed consent once they have been signed. Once patients have been included in the study, the medical monitor will be informed of their inclusion and the randomization envelope will be opened to determine the study group to which they will belong.

Patients who were considered for inclusion but were not admitted to the study will be registered in a patient-log along with the cause for their non-inclusion (lack of informed consent, selection criteria, practical issues, and so on).

Inclusion phase (days −3 or −2): After recording in the case report file (CRF) that the patient meets the selection criteria, the patient's clinical situation will be reviewed and the following information about the medical history will be recorded in the CRF: personal history and anamnesis, including concomitant medication with current dosage and regimen, and complete physical examination which will include height and weight. The patient will also be provided with a card listing basic information about the trial, what to do in the event of severe adverse events and the research team's telephone numbers. The start of the treatment with acenocoumarol will occur on day −3 or −2, according to standard clinical practice. The following dosages will be provided to the researcher as a guideline for the local recommendations: 2 mg a day for patients below 65 years of age and 1 mg a day for older patients or those with risk factors.

Pharmacogenetic specimen collection: During recruitment, a blood sample will be taken from the patient (two tubes of 5 mL EDTA K3) and will be sent by messenger to the laboratory where the patient's genotype will be determined.

The samples do not require special conservation or transportation conditions. In the event that the messenger collects the sample the day after extraction, it is recommended that the blood sample be stored in a refrigerator.

The results will be sent by email within 48 hours. The medical monitor will ensure compliance with these deadlines and will solve any logistical problems that may arise.

Individualization phase:

Day 0:

– Control group: The INR will be determined in a capillary blood sample by means of a CoaguCheck® portable coagulometer (Roche FarmaSA, Madrid, SPAIN), and the acenocoumarol dosage will be determined according to standard clinical practice.

– Intervention group: At this time, the results of the genotyping will have returned and the acenocoumarol dose will be calculated according to the developed algorithm.

Days 3 and 7 ± 1: The INR will be determined (capillary blood sample analyzed with a portable coagulometer) in both groups, and the acenocoumarol dosage will be adjusted if necessary. Control group patients will follow the dosage indications according to the standard clinical practice and visits will be scheduled for day 3, day 7 ± 1 and day 15 ± 3, depending on the length of the hospital stay. Patients included in the intervention group will have their acenocoumarol regimen readjusted according to the developed algorithm and will be scheduled for visits on day 3, 7 ± 1 and 15 ± 3, depending on the length of the hospital stay.

INR measurements will be performed on both groups and additional visits deemed appropriate for good control of the anticoagulation will be arranged. Any changes made to the dosage and all INR measurements will be recorded in the CRF and the medical records.

Visit days 15 ± 3, 30 ± 5, 60 ± 7 and 90 ± 7: During each visit, and after determining the INR (capillary blood sample analyzed with a portable coagulometer), the dosage will be adjusted. The patient's next scheduled visit will be arranged, and an additional visit will be scheduled if necessary. Adverse events will be recorded during all visits, as contained in “Adverse events” section of this protocol. During the final visit, a complete physical examination will also be performed.

### Concomitant medication

– After the diagnosis, as recommended by international guidelines on the treatment of VTE, all patients (both the control and experimental groups) will receive low molecular weight heparins (LMWH) for at least seven days.

– All other medication that the patient is taking will be recorded in their medical history and CRF, as well as any changes they present during the course of the study. We will record the brand name of the medication, the active ingredient, dosage schedule, start or change in treatment, end of treatment and whether there has been any resolution of the problem that caused the start or change of treatment.

As is standard practice, the study's medical researchers will take into account this change in medication when adjusting the acenocoumarol dosage for both the experimental and control group.

### Randomization

The randomization was performed using a masked randomization scheme, in a 1:1 ratio, in blocks of four patients and stratified by center. Based on this scheme, randomization envelopes were created for each center. The patient's code was written on the outside of these envelopes (center and patient codes) and inside the opaque sealed envelope was a card indicating the study group to which the patient was assigned. The envelopes and card were stored in the investigator file.

### Masking

The study is single blind, and under no circumstance does the patient know the group to which they have been assigned. The medical researchers are unaware of the randomization scheme. Although there is a risk of unmasking the patient, and the lack of masking for the physician may affect the evaluation variables, we believe that total masking is not feasible in a study using a pragmatic approach.

### Genotyping

In the recruitment visit, two 3 ml tubes of blood will be collected from all participants for genotyping of CYP2C9*2 (rs1799853), CYP2C9*3 (rs1057910), *VKORC1* (*−*1639 G *→* A = rs9923231), *CYP4F2* (rs2108622), and *APOE* (8016 C *→* T = rs7412), using a previously developed multiplex technique [28]. Measurements will be performed in a centralized manner at the Laboratory of Population Genetics of the Department of Forensics at the Universidad Complutense de Madrid.

### Safety procedures and monitoring of adverse events

#### Anticoagulation monitoring

The INR (International Normalized Ratio) will be determined using a portable coagulometer (CoaguCheck®, Roche FarmaSA, Madrid, SPAIN)) during the visits on days 0, 3, 7, 15, 30, 60 and 90, to verify that the patients are properly anticoagulated and to make the necessary dose adjustments. Dosing adjustments will be performed according to standard clinical practice. For the experimental group, the genetic results will also be taken into account.

If clinically necessary, additional INR measurements and visits will be performed according to standard clinical practice.

#### Laboratory safety determinations

A laboratory analysis including hemogram, biochemistry and coagulation will be performed on the second visit (day 0) and on the eighth or final visit (day 90 ± 7). A hard copy of the results must be signed and dated by the applicant researcher. Results with values outside the normal range will be evaluated by the researching physician who will indicate whether they are clinically relevant or not. Only clinically relevant results will be recorded in the CRF.

Any additional analysis during any of the study visits, requested either by the researcher or other physician, will be recorded in the medical history and CRF along with the reason for the request. A copy of the analysis will be attached to the patient's medical record.

### Adverse events

Any adverse event (not necessarily related to the use of acenocoumarol) will be recorded in the case report form and will be defined as a serious adverse event if it places the patient's life in danger and/or requires or prolongs hospitalization (for example, a major hemorrhagic event).

Hemorrhages will be categorized as major or minor, according to the classification for hemorrhagic events of the International Society on Thrombosis and Haemostasis (ISTH) [29]. We will use the Naranjo *et al*. algorithm [30] to determine any causal relationship between the adverse event and the study treatment.

### Withdrawal criteria

The patient can discontinue their participation in the study at any time. The research doctor, in his or her opinion or judgment, may also withdraw a patient from the study if required by the patient's clinical situation or if the patient does not comply with the protocol. The onset of chronic disease during the study will result in exclusion of the subject. The continuation of the patient in the study after the onset of acute disease will be at the discretion of the researcher, taking into account the patient's safety and the influence of the disease and/or its treatment on the evaluation variables.

### Sample size calculation

We need 97 subjects per group to complete the study in order to detect an absolute difference of 20% in the number of patients who are within the therapeutic range on the seventh day after starting the treatment. For this calculation, we have set an alpha error of 0.05 and a beta error of 0.2, and we assume that 40% of the patients will be within the desired INR, and expect this to increase to 60% in the experimental group. Given the risk of drop-out, the number of patients per group will increase by 20%, placing the recruitment objective at 120 patients per group.

According to Caraco *et al*. [31], pharmacodynamic stabilization with warfarin in the standard control group was achieved in 40.2 ± 21 days. For a reduction in ten days in the experimental group and alpha and beta errors of 0.05 and 0.2 respectively, the required number of patients per group will be 70.

### Statistical analysis

All recruited patients will be included in the main analysis, following an ‘intention to treat’ (ITT) strategy. For the ITT analysis, patients lost to follow up will be classified in the following way: 1) patients lost before the seventh day will be considered to be outside the therapeutic range; 2) patients lost before the last visit and that have not reached the stable INR will be considered not to be in stable dosing; 3) all lost visits, the INR will be accounted as being out of the therapeutic range. In addition, we will perform a per protocol analysis on all patients who can be monitored for the three month period planned for the study and who have not missed more than one intermediate follow-up visit.

To compare the analysis variables between both groups, a contrast test of the hypothesis will be appropriate based on the normality of the sample (Kolmogorov-Smirnov test), with a Cox regression for dichotomous variables or Kaplan-Meier curves for representing results whenever possible. A regression model (linear or logistic, as appropriate) will be performed when necessary to correct for possible confounding factors. To determine the difference between the two groups in the proportion of time within the therapeutic range, we will use the linear interpolation method proposed by Rosendaal *et al*. [27]. Finally, we will perform a regression model stratified by center. The value *P* <0.05 will be established as statistically significant, adjusting for multiple comparisons whenever necessary.

The cost-effectiveness analysis will be performed according to a parallel and independent protocol, with special consideration of the direct costs associated with the health care of these patients.

### Ethical and legal considerations

The researchers will adhere strictly to the provisions of this protocol and will complete the case report forms. The study will be performed according to the recommendations for clinical studies and the evaluation of drugs in humans, as contained in the Declaration of Helsinki (revised in successive world assemblies) and in the current Spanish and European legislation on clinical studies and patient data confidentiality. The study will follow the principles of Good Clinical Practice. This study has been approved by the Clinical Research Ethics Committee of the Hospital Universitario La Paz (Madrid, Spain) and by the Spanish Agency of Medication and Health Products and has been registered in Eudra CT (Eudra CT: 2009-016643-18).

## Discussion

Anticoagulant therapy with acenocoumarol is associated with a high incidence of hemorrhagic complications as well as therapeutic failure (new thrombosis) [1]. These complications are the result of the high interindividual variability in the doses of acenocoumarol required to achieve stable anticoagulation and the time required to reach the steady state of the drug, which according to Caraco *et al*. [31], may require 40.27 days (95% CI, 35.9 to 44.6 days) for warfarin.

One of the strategies for reducing this time and thereby increasing both the efficacy and safety of these drugs is the use of dosing algorithms that include demographic, clinical and genetic variables. These algorithms, several of which have been published for warfarin [13-22] and only three of which have been published for acenocoumarol [23-25], must be validated through randomized clinical trials in order to demonstrate their efficacy and evaluate their effectiveness, as well as the feasibility of their implementation in standard clinical practice. There are two on-going clinical trials, one in the USA using warfarin, and another in Europe (EU-PACT) evaluating algorithms for acenocoumarol, phenprocoumon and warfarin [26].

Our clinical trial has several relevant differences from the studies underway in the USA (in addition to the anticoagulant used) and in Europe. Firstly, our algorithm is the only one to consider the *CYP4F2* and ApoE genes, in addition to the more common demographic, clinical (age, BMI and concomitant treatment with amiodarone and metabolic inducers) and genetic (*CYP2C9* and *VKORC1*) variables. Secondly, the study population only includes patients with VTE. These patients, analyzed as a whole, tend to be younger (although they have a wider age range) and have fewer concurrent conditions [32]. Clearly, this implies the theoretical advantages of greater homogeneity of the included sample and a smaller sample size required. In contrast, the extrapolation of results to patients with atrial fibrillation or valve replacements may be compromised.

Another aspect to consider is that the usefulness of the genetic determination may be more important for patients with VTE, given that it is important to achieve a therapeutic range in the shortest time possible because the risk of progression, recurrence and death due to PTE is greater in the first weeks after the diagnosis [33-35]. Furthermore, the risk of bleeding is also greater at the beginning of treatment; 62.5% of hemorrhages occur during the first 15 days of treatment and 79.2% occur during the first month (median, 11 days). In addition, approximately 50% of patients in therapy with anti-vitamin K have an INR >3 [36].

In addition, the improved variables that reflect appropriate anticoagulant control (time within the appropriate INR, the number of INRs in the range, and so on) could decrease the visits required for necessary monitoring and therefore improve the patient's quality of life and the cost of the entire process. Finally, the time required with VTE to properly adjust the oral anticoagulant dose (with a frequency greater than one month) is a substantial part of the total recommended treatment (three to six months). However, these two aspects are less relevant in the case of atrial fibrillation and valve replacement, in which the short-term risk of ineffectiveness is much lower and anticoagulation is required for life.

We selected appropriate anticoagulant control as the primary objective, including variables such as the percentage of patients within the therapeutic range in the short, medium and long-term or the time required to achieve control. This represents a limitation compared with the use of hard clinical variables (hemorrhages, recurrence of VTE, the progression of PTE and/or the presence of post-phlebitic syndrome). The decision to establish this primary objective was based on logistical reasons and due to its validity as a subrogated endpoint. One of the logistical reasons was the need to perform the genotypic determinations in a centralized laboratory and within a short period of time (at most two days). Therefore, the recruitment centers had to be concentrated in the city of Madrid or its suburbs. This in turn places limitations on the number of patients who could be recruited given that the study population is limited to that of the centers. Similarly, for financial reasons, the recruitment of several thousand patients was out of our reach. There is compelling evidence that maintaining an INR within the therapeutic range is a valid objective of anticoagulant therapy and is linked with clinical end-points of interest, and as such it is used routinely as a control treatment [37-40]. When implementing pharmacogenetic techniques and dosing algorithms, it is important to consider that local issues related to the differences in the type of study population, genetic differences and differences in the healthcare system are of great importance.

In summary, the proposed clinical trial will constitute an important proof of the usefulness of dosing algorithms that include pharmacogenetic variables.

## Trial status

This protocol was authorized on 18 March 2010 by the Spanish Medicine Agency in its initial version, with subsequent changes. The protocol described here corresponds to version 5. The Ministry of Health granted partial support for the implementation of the trial (project TRA-010), and CAIBER (Spanish Platform for Clinical Trials) was responsible for the monitoring and coordination of the trial and also partially financed the implementation of the trial. The first patient was recruited in 21 March 2011 and currently, a total of 85 patients have been included. We plan to continue recruitment until December 2012.

## APPENDIX: Trial Investigation Group

• Trial Coordination: Carcas Sansuán, Antonio J.

• Clinical Researchers:

○ Hospital Universitario La Paz:

▪ Department of Clinical Pharmacology: Alberto M. Borobia, Jesús Frías Iniesta, Elena Ramírez García, Marta Velasco, Hoi Y. Tong.

▪ Emergency Department: Manuel Quintana Díaz, Ana Martínez Virto, Sara Fabra Cadenas, Manuel González Viñolis.

▪ Department of Internal Medicine: Carmen Fernández-Capitán, Alicia Lorenzo, Maria Ángeles Rodríguez Dávila.

○ Hospital Universitario La Princesa:

▪ Department of Clinical Pharmacology: Francisco Abad-Santos, Dolores Ochoa, Igone Marrodan, Isabel Moreno, Manuel Román, Carmen Verge.

▪ Department of Internal Medicine: Carmen Suarez Fernández, Nuria Ruiz-Giménez.

○ Hospital Infanta Sofía:

▪ Department of Internal Medicine: Jorge Gómez Cerezo, Olga Madridano, Mar Martín del Pozo.

○ Fundación Jiménez Díaz:

▪ Department of Hematology: Pilar Llamas Sillero.

○ Hospital 12 de Octubre:

▪ Department of Internal Medicine: Agustín Blanco Echevarría.

• Pharmacogenetic determinations:

○ Universidad Complutense de Madrid: Eduardo Arroyo Pardo, Ana Maria López Parra, Carlos Baeza.

• Study monitoring:

○ CAIBER: Spanish Platform for Clinical Trials.

## Abbreviations

ApoE: Apolipoprotein E; CAIBER: Spanish platform for clinical trials; CI: Confidence interval; CRF: Case report form; CYP2C9: Cytochrome P450 2C9; CYP4F2: Cytochrome P450 4 F2; DVT: Deep venous thrombosis; EU-PACT: European pharmacogenetics of anticoagulant therapy; INR: International normalized ratio; ISTH: International society on thrombosis and hemostasis; ITT: Intention to treat; LMWH: Low molecular weight heparins; PTE: Pulmonary thromboembolism; VKORC1: Complex 1 of the vitamin K epoxide reductase; VTE: Venous thromboembolic.

## Competing interests

The authors have no conflicts of interest relevant to this article to disclose.

## Authors’ contributions

AJC conceptualized and designed the clinical trial, drafted the initial manuscript, and approved the final manuscript as submitted. AJC is the Trial Coordinator. AMB conceptualized and designed the clinical trial, drafted the initial manuscript, and approved the final manuscript as submitted. MV contributed to the design of the clinical trial, has reviewed the manuscript, and approved the final manuscript as submitted. FAS contributed to the design of the clinical trial, reviewed the manuscript, and approved the final manuscript as submitted. FAS is the Principal Investigator of the Clinical Pharmacology Department of La Princesa Hospital. MQD has reviewed the manuscript, and approved the final manuscript as submitted. MQD is the Principal Investigator of the Emergency Department of La Paz University Hospital. CFC contributed to the design of the clinical trial, reviewed the manuscript, and approved the final manuscript as submitted. CFC is the Principal Investigator of the Internal Medicine Department of La Paz University Hospital. NRG contributed in the design of the clinical trial, reviewed the manuscript, and approved the final manuscript as submitted. OM contributed to the design of the clinical trial, reviewed the manuscript, and approved the final manuscript as submitted. PLS has reviewed the manuscript, and approved the final manuscript as submitted. PLS is the Principal Investigator of the Hematology Department of Fundacion Jimenez Diaz Hospital. All authors read and approved the final manuscript.

## Financial disclosure

This clinical trial is possible thanks to a grant from Ministerio de Sanidad, Servicios Sociales e Igualdad (TRA-010) del Gobierno de España and to the technical and financial support of CAIBER (Plataforma Española de Ensayos Clínicos) and IdiPAZ (Instituto de Investigación del Hospital Universitario La Paz).
